# Associations between Cerebrovascular Function and the Expression of Genes Related to Endothelial Function in Hormonal Migraine

**DOI:** 10.3390/ijms25031694

**Published:** 2024-01-30

**Authors:** Jemima S. A. Dzator, Robert A. Smith, Kirsten G. Coupland, Peter R. C. Howe, Lyn R. Griffiths

**Affiliations:** 1 School of Biomedical Sciences and Pharmacy, University of Newcastle, Newcastle, NSW 2308, Australiapeter.howe@newcastle.edu.au (P.R.C.H.); 2 School of Pharmacy and Medical Sciences, Griffith University, Gold Coast, QLD 4222, Australia; 3 Genomics Research Centre, Centre for Genomics and Personalised Health, Queensland University of Technology, Brisbane, QLD 4059, Australia; 4 Hunter Medical Research Institute, New Lambton Heights, NSW 2305, Australia; 5 Adelaide Medical School, University of Adelaide, Adelaide, SA 5005, Australia; 6 Centre for Health Research, University of Southern Queensland, Raceview, QLD 4350, Australia

**Keywords:** cerebrovascular, endothelial function, gene expression, hormonal migraine, migraine, resveratrol

## Abstract

There is evidence to suggest that hormonal migraine is associated with altered cerebrovascular function. We aimed to investigate whether the expression of genes related to endothelial function in venous blood (1) might influence cerebrovascular function, (2) differs between hormonal migraineur and non-migraineur women, and (3) changes following resveratrol supplementation. This study utilised data obtained from 87 women (59 hormonal migraineurs and 28 controls) where RNA from venous blood was used to quantify gene expression and transcranial Doppler ultrasound was used to evaluate cerebrovascular function. Spearman’s correlation analyses were performed between gene expression, cerebrovascular function, and migraine-related disability. We compared the expression of genes associated with endothelial function between migraineurs and non-migraineurs, and between resveratrol and placebo. The expression of several genes related to endothelial function was associated with alterations in cerebrovascular function. Notably, the expression of *CALCA* was associated with increased neurovascular coupling capacity (*p* = 0.013), and both *CALCA* (*p* = 0.035) and *VEGF* (*p* = 0.014) expression were associated with increased cerebral blood flow velocity in the overall study population. Additionally, *VCAM1* expression correlated with decreased pulsatility index (a measure of cerebral arterial stiffness) (*p* = 0.009) and headache impact test-6 scores (*p* = 0.007) in the migraineurs. No significant differences in gene expression were observed between migraineurs and controls, or between placebo and resveratrol treatments in migraineurs. Thus, altering the expression of genes related to endothelial function may improve cerebrovascular function and decrease migraine-related disability.

## 1. Introduction

Hormonal migraine describes migraines that occur within a few days of the onset of menstruation. Estimated to afflict one in two women with migraine, hormonal migraines are triggered by the rapid decrease of estrogen concentrations that occur prior to the onset of menstruation [1,2]. As estrogen contributes to healthy vascular function, it is possible that underlying deficits of cerebrovascular function in hormonal migraineurs are exacerbated by the rapid decrease of estrogen [3,4]. Previously, we reported cerebrovascular function to be poorer in hormonal migraineurs than in controls, as evidenced by lower neurovascular coupling capacity and lower resting blood flow velocity in the middle cerebral artery [5]. Furthermore, Tietjen et al. reported that migraineurs (particularly premenopausal migraineurs) have endothelial dysfunction, as evidenced by elevated concentrations of inflammatory markers such as C-reactive protein and lower nitrate and nitrite levels compared to controls; this may reflect the involvement of cerebrovascular dysfunction in the pathophysiology of hormonal migraine [6]. Building on these findings, we previously investigated whether supplementing the diet with resveratrol (a phytoestrogen with vasoactive properties) for three months could improve cerebrovascular function in hormonal migraineurs [7,8]. We reported some modest alterations in cerebrovascular function following resveratrol supplementation that might have resulted from altered expression of endothelial genes of interest in hormonal migraineurs, but this remains to be directly examined [7].

In addition to the roles that hormonal fluctuations and vascular function may play in the pathogenesis of hormonal migraine, recent evidence suggests that some women may be genetically predisposed to hormonal migraine. Please note that in this present study, we use the term “hormonal migraine” rather than “menstrual migraine” to describe our participants, as our enrolment criteria utilised a slightly modified version of the criteria for menstrual migraine as defined by the International Classification of Headache Disorders-3. In a study conducted by Rodriguez-Acevedo et al. [9], two SNPs, rs3093664 and rs9371601 in tumour necrosis factor (*TNF*) and synaptic nuclear envelope protein-1 (*SYNE 1*) genes respectively, were found to be significantly associated with menstrual migraine. Although they found no differences in gene expression between migraineurs and non-migraineur controls, they observed correlations between the expressions of *TNF*, *SYNE 1*, estrogen receptor 1, and progesterone receptor genes which differed between follicular and luteal phases of the menstrual cycle. Some of these correlations in gene expression were also found to be weaker or absent in controls.

There is evidence that both genetic factors and reduced cerebrovascular function are associated with hormonal migraine; however, it is not currently clear whether these deficits in cerebrovascular function in hormonal migraineurs are related to expression of genes associated with endothelial function. It remains to be investigated whether the expression of genes related to endothelial function is associated with altered cerebrovascular function in hormonal migraineurs, or whether there are differences in the expression of genes related to endothelial function between hormonal migraineurs and controls. Furthermore, no previous study has investigated whether resveratrol supplementation can alter the expression of genes related to endothelial function in hormonal migraineurs. Therefore, the main aims of this post-hoc study are to investigate whether (1) the expression of genes related to endothelial function in venous blood is associated with cerebrovascular function, (2) the expression of genes related to endothelial function in venous blood differs between hormonal migraineurs and controls, and (3) resveratrol supplementation can alter the expression of genes related to endothelial function in hormonal migraineurs.

## 2. Results

Baseline characteristics of participants can be seen in Table 1. There were no significant differences in baseline characteristics between hormonal migraineurs and controls.

**Aim 1.**  *Associations between gene expression, cerebrovascular function, migraine-related disability, and quality of life.*

**Figure 1:**
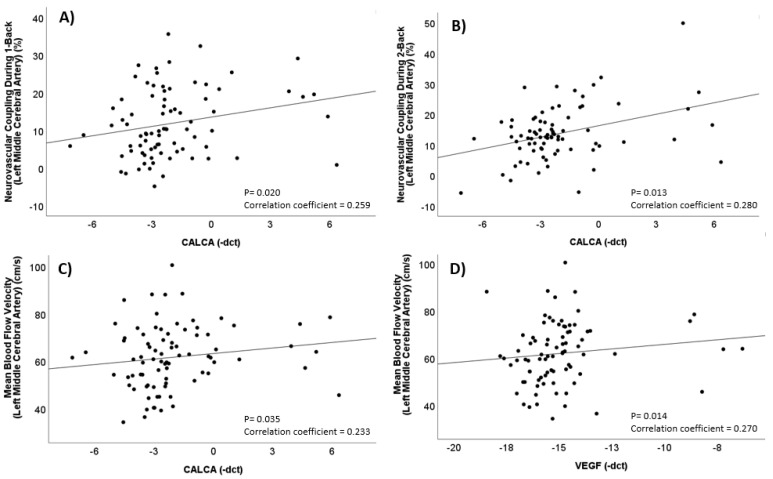
Correlations between *CALCA*, *VEGF*, and measures of cerebrovascular function in the left middle cerebral artery. (**A**) Spearman correlation between *CALCA* (−ΔCt) and neurovascular coupling capacity during the 1-back task, (**B**) Spearman correlation between *CALCA* (−ΔCt) and neurovascular coupling capacity during the 2-back task, (**C**) Spearman correlation between *CALCA* (−ΔCt) and mean blood flow velocity, and (**D**) Spearman correlation between *VEGF* (−ΔCt) and mean blood flow velocity.

Higher expression of *CALCA* was associated with higher neurovascular coupling capacity during the 1-Back (Spearman’s correlation coefficient = 0.259; *p* = 0.020) and 2-Back (Spearman’s correlation coefficient = 0.280; *p* = 0.013) tasks, and higher mean blood flow velocity (Spearman’s correlation coefficient = 0.233; *p* = 0.035) in the left middle cerebral artery when baseline correlation analyses were performed without taking the menstrual phase of the sample into account (see Figure 1). Additionally, higher expression of *VEGF* was associated with mean blood flow velocity in the left middle cerebral artery (Spearman’s correlation coefficient = 0.270; *p* = 0.014) in the total population, and increased expression of *IL1B* was associated with a higher Migraine Disability Assessment score (Spearman’s correlation coefficient = 0.329; *p* = 0.012) (see Supplementary Figure S1). 

Several additional correlations were identified when analysed according to the menstrual phase of the sample (see Supplementary Tables S1–S3). In the follicular phase, higher *CALCA* expression was associated with higher mean blood flow velocity (Spearman’s correlation coefficient = 0.397; *p* = 0.050) and higher neurovascular coupling capacity during the 1-Back task (Spearman’s correlation coefficient = 0.450; *p* = 0.021) in the right middle cerebral artery. Additionally, higher expression of *VCAM1* was found to be associated with lower migraine severity (Spearman’s correlation coefficient = −0.559; *p* = 0.010) and headache impact test-6 scores (Spearman’s correlation coefficient = −0.596; *p* = 0.007).

In the luteal phase, higher *NOS3* expression was associated with higher mean blood flow velocity (Spearman’s correlation coefficient = 0.615; *p* = 0.044) in the left middle cerebral artery. Higher *VCAM1* expression was associated with a lower pulsatility index (Spearman’s correlation coefficient = −0.743; *p* = 0.009) in the right middle cerebral artery.

Mid-cycle, *VEGF* expression correlated with resting mean blood flow velocity (Spearman’s correlation coefficient = 0.421; *p* = 0.036) in the left middle cerebral artery, and *ICAM1* expression correlated with the pulsatility index (Spearman’s correlation coefficient = 0.419; *p* = 0.030). After correcting for multiple comparisons, there were no significant correlations between the expression of genes related to endothelial function and measures of cerebrovascular function, migraine-related disability, or migraine-related quality of life, regardless of whether phase was taken into account.

**Aims 2 and 3.**  *Gene expression between hormonal migraineurs and controls, and between baseline and resveratrol-treated samples.*

No significant differences in gene expression were detected between hormonal migraineurs and controls (see Supplementary Figure S2), nor between any of the treatment phases for the active treatment samples in hormonal migraineurs (see Supplementary Figure S3). Given that Rodriguez-Acevedo et al. previously observed that the different phases of the menstrual cycle can affect expression relationships between genes [9], we also undertook an analysis taking account of the phase of the menstrual cycle in which the samples were collected, i.e., luteal, follicular, or mid-phase. This analysis also showed no significant difference for any gene between controls and hormonal migraineurs.

## 3. Discussion

In this post-hoc analysis, we found that the expression of several genes known to be related to vascular endothelial function was associated with variations in cerebrovascular function, migraine-related disability, and migraine-related quality of life in hormonal migraineurs. The expressions of *CALCA* and *VEGF* were generally associated with increased cerebral blood flow velocity and neurovascular coupling capacity regardless of menstrual phase or migraine status (i.e., hormonal migraineur or control). In terms of migraine-related disability, an association between *IL1B* expression and the Migraine Disability Assessment was found in hormonal migraineurs, regardless of menstrual phase. We also found associations between the expression of *CALCA*, *EDN1*, *IL6*, *NOS3*, *VCAM1*, and *VEGF* and measures of cerebrovascular function and migraine-related disability when menstrual phase (i.e., follicular, mid-cycle, or luteal) was accounted for. To our knowledge, this is the first study to report on associations between the expression of genes related to endothelial vascular function and measures of cerebrovascular function or migraine-related quality of life in hormonal migraineurs.

The associations between *CALCA* gene expression and cerebrovascular function are of particular interest as the *CALCA* gene encodes calcitonin gene-related peptide (CGRP), which is a potent vasodilator and inflammatory mediator that is thought to be involved in migraine pathophysiology [10,11,12]. Additionally, studies have shown CGRP antagonists to be beneficial in reducing the number of monthly migraine days [13,14]. In our total study population, we found higher *CALCA* expression to be associated with higher resting cerebral blood flow velocity, particularly in the follicular menstrual phase. In our study population of hormonal migraineurs and controls, we also found that increasing expression of *CALCA* was associated with increasing neurovascular coupling capacity during a cognitive task. Interestingly, a previous study reported an association between increased CGRP levels and decreased cerebral blood flow velocity. Visocnik et al. reported that infusion of 30 mcg of αCGRP decreased mean blood flow velocity in the middle cerebral artery (*p* < 0.001) and posterior cerebral artery (*p* < 0.001) in 20 healthy subjects, reflecting evidence of CGRP-mediated vasodilation [15]. The relationship between cerebrovascular function and *CALCA* expression seen in our study may be a result of feedback mechanisms signalling for increased *CALCA* expression to counteract higher resting cerebral blood pressure. A few studies have investigated associations between CGRP concentrations and blood pressure, with mixed findings [16,17]. Masuda et al. found plasma CGRP concentrations to be higher in hypertensive patients than controls, whereas Schifter et al. reported no difference in CGRP concentrations between those with hypertension and controls [16,17]. However, when hypertensive participants and controls were analysed as one group, Schifter et al. reported CGRP concentrations to be associated with systolic and diastolic blood pressure [16]. This is of interest as blood pressure can influence blood flow velocity. Blood pressure, a form of potential energy, is converted into blood flow velocity (a form of kinetic energy) [18]. In this present study, we found no difference in blood pressure between hormonal migraineurs and controls. However, it is possible that our findings result from our data being collected in the interictal period, and data collected during a migraine attack may show a difference.

Previous studies suggest that blood pressure may change across the menstrual cycle, particularly around the onset of menstruation [19,20]. However, again, there are mixed findings, with some reporting an increase in blood pressure and others reporting a decrease in blood pressure around the onset of menstruation [19,20,21]. Perhaps changes in blood pressure across the menstrual cycle may influence *CGRP* expression.

There is conflicting evidence regarding whether migraine is associated with hypertension [22,23]. In a systematic review, Wang et al. were unable to conclude whether migraine is associated with hypertension but reported that diastolic blood pressure in migraine tended to be higher than controls [22]. Additionally, in a cross-sectional study of 1134 participants, Zhang et al. found migraine and severe headaches to be associated with hypertension [23]. An association of migraine with high blood pressure may explain why antihypertensive medications such as beta blockers (i.e., propranolol) are widely and effectively used as migraine prophylaxis [24]. In a recently published systematic review and meta-analysis of 60 comparisons, migraine monthly headache days were reduced with the use of the following antihypertensive medications: alpha-blockers, angiotensin-converting enzyme inhibitors, angiotensin receptor blockers, beta blockers, and calcium channel blocks, compared to placebo [25].

We also found higher *VEGF* expression to be associated with higher cerebral blood flow velocity in our population of hormonal migraineurs and controls, regardless of menstrual phase. VEGF is involved in blood vessel proliferation and is induced by hypoxia to promote the growth of blood vessels in hypoxic cells and tissues [26]. VEGF has been shown to increase endothelial nitric oxide synthase, and therefore nitric oxide production [27]. When accounting for menstrual phase, we found higher *VEGF* expression during mid-cycle to be associated with higher neurovascular coupling capacity (cerebrovascular responsiveness to cognitive stimulation) and cerebral blood flow velocity. Perhaps, similarly to *CALCA*, increased expression of *VEGF* may be a regulatory response to increased resting cerebral blood flow, to stabilise overall pressure. Previous studies have shown VEGF inhibitor therapy to be associated with hypertension [28,29]. The incidence of hypertension with VEGF inhibitor therapy has been said to range from 25–90%, with almost all patients experiencing an absolute increase in their blood pressure compared to baseline [28,29]. The *VEGF* expression levels seen in our results may thus be counteracting increased blood pressure from the observed *CALCA* expression levels.

Another gene of particular interest is *VCAM1*, which facilitates the adhesion of leukocytes to endothelial cells, is involved in the inflammatory response, and is a biomarker of endothelial dysfunction [30]. A previous study reported an association between increased levels of *VCAM1* and arterial stiffness [31,32]. Additionally, VCAM1 has been utilised as a marker of arterial stiffness [30,33]. Surprisingly, our baseline correlation analyses revealed a negative correlation between *VCAM1* expression in the luteal phase and stiffness in the right middle cerebral artery. However, it is important to note that previous studies measured serum *VCAM1* levels rather than *VCAM1* mRNA levels in blood cells. Additionally, expression of *VCAM1* negatively correlated with migraine-related disability (headache impact test-6 scores), as well as migraine severity in the follicular phase.

Some studies suggest that increased levels of *VCAM1* may have a protective effect on cardiovascular disease risk. In a longitudinal study, Kunutsor et al. found plasma *VCAM1* levels to be inversely associated with cardiovascular disease [34]. Ulyanova et al. suggested that *VCAM1* may dampen the release of progenitor cells from bone marrow, and with *VCAM1* deletion may come an elevation in the release of immature B-cells and progenitor cells from the bone marrow into the peripheral blood [35]. In a study by Huse et al., it was found that rituximab, which decreases B-cell levels, administered at baseline and day 15 significantly improved endothelial function from baseline and reduced systemic inflammation [36]. Reduced systemic inflammation as a result of decreased B-cell levels may be involved in minimising cardiovascular disease outcomes, as inflammation plays a key role in the development of cardiovascular disease [34,37]. Thus, increased expression of *VCAM1* in peripheral leukocytes, as seen in our results, might represent reduced inflammatory potential, which would be consistent with lower arterial stiffness. As highlighted previously, our baseline correlation analyses did not remain significant after correction for multiple comparisons.

Analysis of the qPCR results indicated that there were no significant differences in gene expression between hormonal migraineurs and non-migraineur controls. This is consistent with previous studies, including that of Rodriguez-Acevedo et al., which found no difference in *TNF* expression levels between menstrual migraineurs and controls [9]. Likewise, Mueller et al. studied levels of IL1B, IL6, and TNFα in the urine of hormonal migraineurs and controls, and found no difference between them, though a sub-set of individuals with no detectable levels of TNFα was identified in their study [38]. While no studies are available specifically addressing the expression of the remaining genes in hormonal migraine, Ibrahimi et al. determined that the significant increase in dermal blood flow response mediated by the product of *CALCA*, calcitonin gene-related peptide, in the menstrual cycle was lost in migraineurs, though no gene expression results were available [39]. The absence of differences in gene expression between hormonal migraineurs and controls in our study may imply that, if any of the genes in our study are involved in hormonal migraine attacks, the effect is not mediated by a steady-state difference in expression and may be mediated by changes in the way the gene pathways function at different points in the menstrual cycle, as in the altered *CALCA* response noted by Ibrahimi et al., but this remains to be directly examined. Alternatively, our small sample size may have affected our ability to identify any significant differences in gene expression between hormonal migraineurs and controls or identify any changes in gene expression following resveratrol supplementation compared to placebo. Future studies should include expanded cohort sizes and, wherever possible, attempt to collect samples in the same phase of the menstrual cycle or, ideally, at all stages, so that any altered response at different times can be effectively detected.

Whilst this study is the first to investigate associations between cerebrovascular function and genes related to endothelial function in hormonal migraineurs, there are a few limitations. Firstly, given that our samples were incubated at room temperature for two to 48 h, it is possible that the gene expression profile changed with increased incubation time. However, longer incubation times could potentially be advantageous, as a previous study indicated higher total RNA yield with increased incubation time [40]. Secondly, our use of only one reference gene, *GAPDH*, may reduce the robustness and precision of our findings, as natural variations in our samples may be unaccounted for. Nonetheless, previous studies have shown *GAPDH* to be a stable reference gene for gene expression analysis in human blood [41,42]. Thirdly, it is possible that the accuracy of RNA concentration measurements obtained using the Nanodrop spectrophotometer may have been affected by potential residual DNA contamination following DNase I treatment. However, it is important to note that the impact of DNA contamination on expression results is likely minimal, as we used relative quantitation methods. Moreover, the use of intron-spanning primers ensured that the PCR fragments derived from RNA and genomic DNA would be different, and the genomic DNA fragment would be too large to be replicated. Fourthly, our study only investigated associations between measures of cerebrovascular function and the expression of genes related to endothelial function, and therefore does not provide information on how systemic vascular function may be linked with the expression of genes associated with endothelial function. Further studies are needed to evaluate whether systemic vascular function is associated with the expression of genes related to endothelial function. Finally, whilst our study provides information on the expression of genes associated with endothelial function, it is important to note that gene expression levels may not accurately reflect the actual level of protein. Therefore, future studies should consider using function analysis techniques such as Western Blot analysis to validate actual protein expression levels.

## 4. Methods and Materials

### 4.1. Study Design and Population

**Figure 2:**
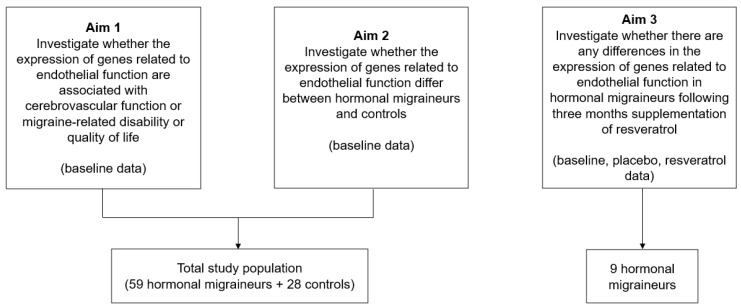
Outline of the aims and study population.

This study comprised data obtained from 87 women (59 hormonal migraineurs and 28 controls) in two studies (a cross-sectional clinical trial [5] and a randomised, double-blind, placebo-controlled, crossover intervention trial [43]) recruited by the Clinical Nutrition Research Centre at the University of Newcastle (see Figure 2). The cross-sectional clinical trial comprised both hormonal migraineurs and controls, whereas the crossover intervention trial included nine hormonal migraineur participants only. Participants in both studies were considered to have hormonal migraine if they fulfilled the criteria for migraines as defined by the International Classification for Headache Disorders [44] and if they suffered from migraines that occurred ±3 days from the onset of their period (or during ovulation for the cross-sectional study) in their three previous menstrual cycles. The inclusion and exclusion criteria for both studies have been described in detail elsewhere [5,43]. Briefly, participants in the placebo-controlled, crossover intervention trial were randomised to take resveratrol (150 mg/day) or placebo capsules twice daily for three months before crossing over to the other treatment arm for another three months. Data were collected from these participants at three time points (month-0 (baseline), month-3, and month-6) [43]. Participants in the cross-sectional clinical trial visited the research centre once and underwent testing to assess their cerebrovascular function, migraine-related disability, and migraine-related quality of life at one time-point [5].

The protocols for both studies were approved by the University of Newcastle’s Human Research Ethics Committee (H-2018-0167 and H-2019-0416) and registered with the Australian and New Zealand Clinical Trials Registry (ACTRN12618001230246 and ACTRN12620000180910). Approval for work conducted at the Queensland University of Technology (QUT) was obtained from the QUT Human Research Ethics Committee (Approval 2000000759). Written informed consent was obtained for all participants from both studies prior to commencement of the trial.

### 4.2. Study Outcomes

Outcomes for both studies were measured when the participant was one-hour fasted, migraine-free, and not menstruating. Study outcomes included cerebrovascular function, migraine-related disability, migraine-related quality of life, and gene expression.

- Cerebrovascular Function

Cerebrovascular function was measured using transcranial Doppler ultrasound. Each participant was fitted with a transcranial Doppler headpiece with one probe fixed to the left and right sides of the temporal region for the bilateral measurement of cerebral blood flow velocity. Cerebral blood flow velocity was measured in the middle cerebral artery for both studies, and in the posterior cerebral artery for the resveratrol intervention study [5,43]. Cerebral blood flow velocity was measured at rest and during both hypercapnic stimulation (inhalation of carbogen gas (95% O_2_ and 5% CO_2_) for three minutes) and cognitive stimulation (neurovascular coupling). The cognitive tests that were completed by the participants have been described in detail elsewhere [5,43]. Cerebrovascular responsiveness to hypercapnia and neurovascular coupling capacity were calculated using the peak percentage increase in cerebral blood flow velocity during hypercapnic stimulation and during cognitive stimulation, respectively.

b. Migraine Disability and Quality of Life

Migraine-related disability was measured using the Headache Impact Test-6™ and Migraine Disability Assessment [45,46]. Migraine-related quality of life was measured using the Migraine-Specific Quality of Life Questionnaire (version 2.1) [47]. Further details about these questionnaires have been described elsewhere [5].

c. Gene Expression Analysis

Approximately 2.5 mL of venous blood was collected in a 9 mL PAXgene Blood RNA tube (QIAGEN, Clayton, VIC, Australia) by a commercial pathology service at the University of Newcastle, Australia. After a minimum of 2 h (but no more than 48 h) at room temperature to ensure blood cell lysis, the collected blood samples were frozen at −80 degrees Celsius until shipment with dry ice to Queensland University of Technology for gene expression analysis. 

Analysis was limited to the following ten genes related to endothelial function and whose proteins are thought to be altered in migraine [48,49,50,51,52,53,54,55,56,57]: calcitonin-related polypeptide alpha (*CALCA*), endothelin 1 (*EDN1*), hypoxia-inducible factor 1, alpha (*HIF1A*), intracellular adhesion molecule 1 (*ICAM1*), interleukin 1 beta (*IL1B*), interleukin 6 beta 2 (*IL6*), nitric oxide synthase 3 (*NOS3*), tumour necrosis factor (*TNF*), vascular cell adhesion molecule 1 (*VCAM1*) and vascular endothelial growth factor A (*VEGFA*). *GAPDH* was utilised as an endogenous control gene to produce −ΔCt and ΔΔCt values.

RNA was extracted using a PAXgene Blood RNA Kit (QIAGEN, Clayton, VIC, Australia) and automated using a QIAcube extraction instrument (QIAGEN), according to manufacturer instructions. Once extracted, RNA was quantitated using a Nanodrop spectrophotometer (ThermoFisher Scientific, Waltham, MA, USA), and transcribed to cDNA using the Super Script IV First-Strand Synthesis System (ThermoFisher Scientific), according to manufacturer instructions. 

Gene expression analysis was undertaken using qPCR with the following final reaction concentrations: 0.83× GoTaq qPCR Master Mix (Promega Corporation, Madison, WI, USA), forward and reverse primers at 0.83 μM, and cDNA equivalent to 30 ng of RNA input per reaction, in a total reaction volume of 12 μL. Thermal cycling and data acquisition was undertaken using a QuantStudio 6 Flex instrument (ThermoFisher Scientific), with thermal cycling parameters of 95 °C for three minutes, followed by 40 cycles of 95 °C for 20 s, 60 °C for one minute, and 72 °C for one minute. Intron-spanning primers for ten specified genes were designed for this study, to attempt to amplify as many gene isoforms as possible, and are outlined in Table 2.

### 4.3. Statistical Analysis

Cerebral blood flow velocity basal and peak values were determined using Table Curve 2D Version 5.01 (SYSTAT Software Incorporated, San Jose, CA, USA 2002). Neurovascular coupling and cerebrovascular responsiveness to hypercapnia were estimated using the smooth data spline estimation function with 10% and 20% Loess curve fitting, respectively.

Gene expression data were analysed using SPSS Release 27.0.1.0, (IBM Corporation, Armonk, NY, USA). qPCR data were analysed using *t*-tests or One Way ANOVA, as appropriate for the comparison, using both ΔCt (difference in threshold cycle between the housekeeping gene and gene of interest) and 2^−ΔΔCt^ values. We calculated 2^−ΔΔCt^ using the average ΔCt values for a sample’s phase of collection. The α value was set to 0.05.

Baseline correlation analyses were performed to identify correlations between cerebrovascular function, migraine-related disability, and migraine-related quality of life with −ΔCt. Spearman’s correlations were used for all analyses, as data were not normally distributed. The α value was set to 0.05 for the baseline correlation analyses. The Benjamini–Hochberg method was applied to each gene to account for multiple comparisons with a false discovery rate level of 0.1.

## 5. Conclusions

In conclusion, several genes related to endothelial function were found to be associated with decreased cerebral arterial stiffness, increased cerebral blood flow velocity, and decreased migraine-related disability or migraine severity in our total study population of hormonal migraineurs and controls, although findings did not remain significant following corrections for multiple comparisons. Based on our findings, it is important that future intervention studies investigate whether improvements of migraine symptomatology and cerebrovascular function are associated with alterations of genes that are related to endothelial function.

## Figures and Tables

**Table 1:** Baseline Participant Characteristics.

	Hormonal Migraineurs (N = 59)	Controls (N = 28)	*p* Value
Age (years) ^a^	38.63 + 1.05	35.89 + 1.83	0.208
Body mass index (kg/m^2^) ^a^	25.58 + 0.63	25.12 + 1.18	0.443
Systolic blood pressure (mmHg) ^b^	116.35 + 1.52	114.61 + 2.52	0.536
Diastolic blood pressure (mmHg) ^b^	70.75 + 1.11	67.68 + 1.56	0.116

^a^ Mann–Whitney U ^b^ Independent *t*-test.

**Table 2:** Primers used in qPCR analysis.

Primer Name	Primer Sequence (5′-3′)	Transcript	Exon
CALCA Forward	TCAGCATCTTGGTCCTGTTG	NM_001741.3	2
CALCA Reverse	CTGCACATAGTCCTGCACCA	NM_001741.3	3
EDN1 Forward	TGGGAAAAAGTGTATTTATCAGCA	NM_001955.5	4
EDN1 Reverse	TTTGACGCTGTTTCTCATGG	NM_001955.5	5
HIF1A Forward	GCTTGGTGCTGATTTGTGAA	NM_001530.4	6
HIF1A Reverse	TTCTGGCTCATATCCCATCA	NM_001530.4	7
ICAM1 Forward	CTTGAGGGCACCTACCTCTG	NM_000201.3	6
ICAM1 Reverse	CATTATGACTGCGGCTGCTA	NM_000201.3	7
IL1B Forward	CTGTCCTGCGTGTTGAAAGA	NM_000576.3	6
IL1B Reverse	ACTGGGCAGACTCAAATTCC	NM_000576.3	7
IL6 Forward	GGCTGAAAAAGATGGATGCT	NM_000600.5	3
IL6 Reverse	GCTCTGGCTTGTTCCTCACT	NM_000600.5	4
NOS3 Forward	TGTCTGCATGGACCTGGATA	NM_000603.5	10
NOS3 Reverse	CACGATGGTGACTTTGGCTA	NM_000603.5	11
VCAM1 Forward	ATGGAATTCGAACCCAAACA	NM_001078.4	6
VCAM1 Reverse	CCTGGCTCAAGCATGTCATA	NM_001078.4	7
VEGF Forward	CCCACTGAGGAGTCCAACAT	NM_001025366.3	3
VEGF Reverse	TGCATTCACATTTGTTGTGC	NM_001025366.3	4
TNF Forward	GACAAGCCTGTAGCCCATGT	NM_000594.4	3
TNF Reverse	GAGGTACAGGCCCTCTGATG	NM_000594.4	4
GAPDH Forward	CGACCACTTTGTCAAGCTCA	NM_002046.7	8
GAPDH Reverse	GGTGGTCCAGGGGTCTTACT	NM_002046.7	9

## Data Availability

The data presented in this study are available on request from the corresponding author.

## Supplementary Materials

The following supporting information can be downloaded at: https://www.mdpi.com/article/10.3390/ijms25031694/s1.
