# Supplementary material for: Associations between Cerebrovascular Function and the Expression of Genes Related to Endothelial Function in Hormonal Migraine
Source: Int J Mol Sci. 2024 Jan 30;25(3):1694. doi: 10.3390/ijms25031694 (PMC10855027; doi:10.3390/ijms25031694)
Supplement: Supplementary file 1 [file ijms-25-01694-s001.zip › ijms-2793897-supplementary.pdf]

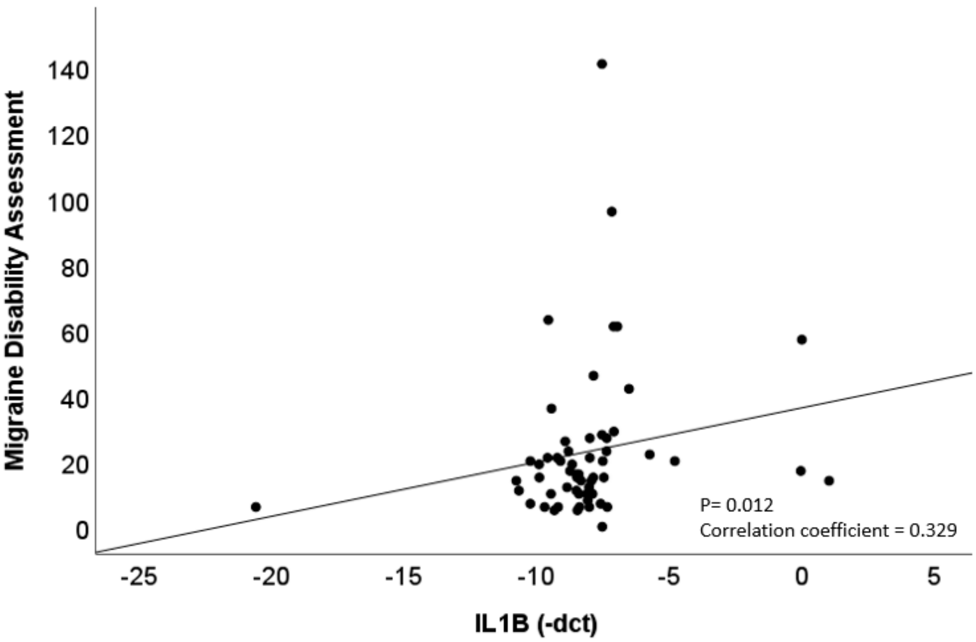

Supplementary Figure S1. Baseline Correlation Analysis for Samples Collected in the Follicular Phase.

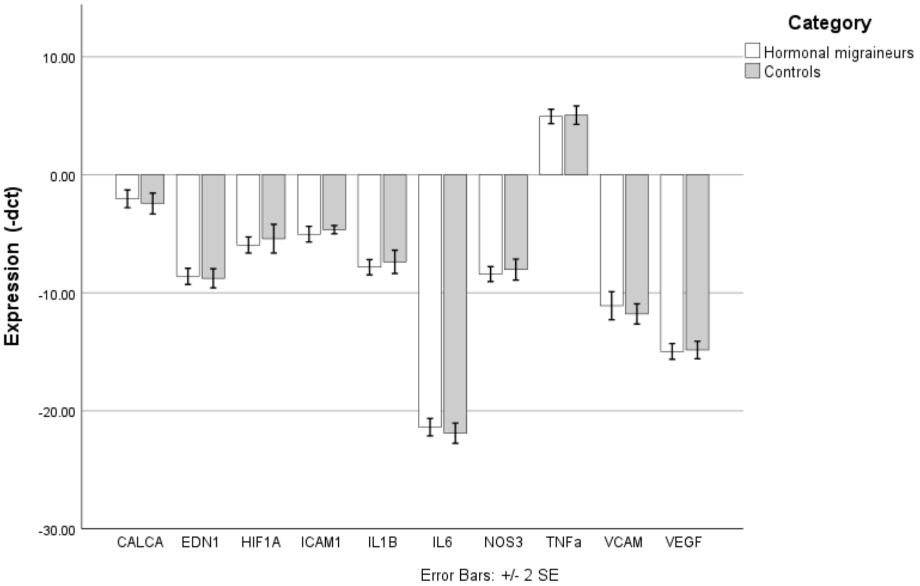

Supplementary Figure S2. Bar Graph Showing a Comparison of Gene Expression (ΔCt) between Hormonal Migraineurs (n = 59) and Controls (n = 28).

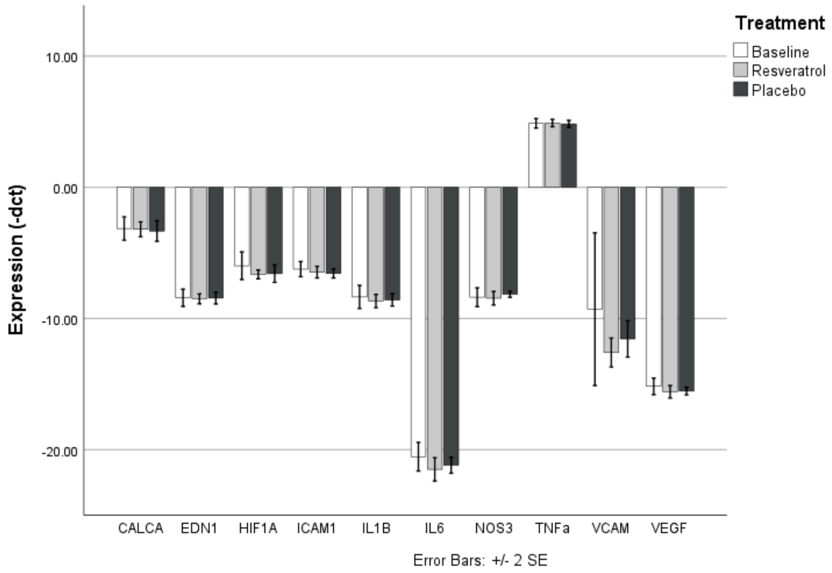

Supplementary Figure S3. Bar Graph Showing a Comparison of Gene Expression (ΔCt) between Baseline (n = 9), Resveratrol (n = 9), and Placebo (n = 9) Treatment Phases.

Supplementary Table S1. Baseline Correlation Analysis for Samples Collected in the Follicular Phase.

	Gene	Correlation co-efficient	Significance	N
**Mean Blood Flow Velocity (Right MCA) (cm/s)**	CALCA	0.397	0.05	25[a]
**Neurovascular Coupling 1-Back (Right MCA) (%)**	CALCA	0.450	0.021	26[b]
**Migraine Severity†**	EDN1	-0.467	0.038	20[c]
**Migraine Severity†**	VCAM1	-0.559	0.01	20[c]
**Headache Impact Test-6**	VCAM1	-0.596	0.007	19[c]

Spearman's correlation analysis. [a] Population comprising 20 hormonal migraineurs and five controls. [b] Population comprising 21 hormonal migraineurs and five controls. [c] Population comprising hormonal migraineurs ONLY. †This is Migraine Disability Assessment Item B.

Supplementary Table S2. Baseline Correlation Analysis for Samples Collected in the Luteal Phase

	Gene	Correlation co-efficient	Significance	N
**Mean Blood Flow Velocity (Left MCA) (cm/s)**	NOS3	0.615	0.044	11[a]
**Neurovascular Coupling 2-Back (Left MCA) (%)**	IL6	0.618	0.043	11[b]
**Neurovascular Coupling 2-Back (Right MCA) (%)**	IL6	0.601	0.039	12[c]
**Neurovascular Coupling 2-Back (Right MCA) (%)**	VCAM1	-0.691	0.019	11[b]
**Neurovascular Coupling 2-Back (Left MCA) (%)**	VEGF	0.62	0.042	11[b]
**Resistive Index (Right MCA)**	VCAM1	-0.733	0.01	11[b]
**Pulsatility Index (Right MCA)**	VCAM1	-0.743	0.009	11[b]

**Pulsatility Index (Right MCA)**	TNF	-0.606	0.048	11[a]
**Resistive Index (Right MCA)**	TNF	-0.629	0.038	11[a]

Spearman's correlation analysis. [a] Population comprising 10 hormonal migraineurs and one control. [b] Population comprising nine hormonal migraineurs and two controls. [c] Population comprising 10 hormonal migraineurs and two controls.

Supplementary Table S3. Baseline Correlation Analysis for Samples Collected in the Mid-Cycle Phase

	**Gene**	**Correlation co-efficient**	**Significance**	**N**
**Mean Blood Flow Velocity (Left MCA) (cm/s)**	VEGF	0.421	0.036	25[a]
**Pulsatility index (Right MCA)**	ICAM1	0.419	0.03	27[b]
**Migraine Disability Assessment**	IL6	0.528	0.029	17[c]

Spearman's correlation analysis. [a] Population comprising 16 hormonal migraineurs and nine controls. [b] Population comprising 16 hormonal migraineurs and 11 controls. [c] Population comprising hormonal migraineurs ONLY.
